# Mitochondrial Dysfunctions in Type I Endometrial Carcinoma: Exploring Their Role in Oncogenesis and Tumor Progression

**DOI:** 10.3390/ijms19072076

**Published:** 2018-07-17

**Authors:** Clara Musicco, Gennaro Cormio, Vito Pesce, Vera Loizzi, Ettore Cicinelli, Leonardo Resta, Girolamo Ranieri, Antonella Cormio

**Affiliations:** 1 CNR–Institute of Biomembranes, Bioenergetics and Molecular Biotechnologies (IBIOM), Via Amendola 165/A, 70126 Bari, Italy; c.musicco@ibiom.cnr.it; 2 Department of Biomedical Sciences and Medical Oncology, University of Bari, P.zza Giulio Cesare, 70124 Bari, Italy; gennaro.cormio@uniba.it (G.C.); vera.loizzi@uniba.it (V.L.); Ettore.cicinelli@uniba.it (E.C.); 3 Gynecologic Oncology Unit, National Cancer Center “Giovanni Paolo II”, Viale Orazio Flacco, 65, 70124 Bari, Italy; 4 Department of Biosciences, Biotechnologies and Biopharmaceutics, University of Bari, Via Orabona, 4, 70125 Bari, Italy; vito.pesce@uniba.it; 5 Department of Pathology, University of Bari, Piazza Giulio Cesare 70124 Bari, Italy; leonardo.resta@uniba.it; 6 Interventional Radiology Unit with Integrated Section of Translational Medical Oncology, National Cancer Centre ″Giovanni Paolo II″, Viale Orazio Flacco, 65, 70124 Bari, Italy; giroran@tiscali.it

**Keywords:** endometrial cancer, mtDNA mutations, deficit of complex I, antioxidant response, mitochondrial biogenesis, mitochondrial dynamics, mitophagy

## Abstract

Type I endometrial cancer (EC) is the most common form of EC, displaying less aggressive behavior than type II. The development of type I endometrial cancer is considered a multistep process, with slow progression from normal endometrium to hyperplasia, the premalignant form, and endometrial cancer as a result of an unopposed estrogenic stimulation. The role of mitochondria in type I EC tumor progression and prognosis is currently emerging. This review aims to explore mitochondrial alterations in this cancer and in endometrial hyperplasia focusing on mitochondrial DNA mutations, respiratory complex I deficiency, and the activation of mitochondrial quality control systems. A deeper understanding of altered mitochondrial pathways in type I EC could provide novel opportunities to discover new diagnostic and prognostic markers as well as potential therapeutic targets.

## 1. Introduction

Mitochondria are double membrane organelles that supply ATP for key cellular processes in all eukaryotic cells, through the oxidative phosphorylation system (OXPHOS), thus functioning as the fulcrum of cellular homeostasis. In addition, mitochondria are primary sources of reactive oxygen species (ROS), and regulate intracellular calcium, apoptosis, signal transduction and redox balance [1,2,3].

Mitochondria contain mitochondrial DNA (mtDNA), a circular, double-stranded DNA of approximately 16569 bp [4]. mtDNA is inherited exclusively from the mother [5,6]. The number of mtDNA copies per cell varies (about 2–4000); this feature is called polyploidy [5]. Moreover, the mtDNA molecules may be all the same type (homoplasmy), wild-type or mutant, or may be of different types (heteroplasmy) in cells or tissues.

MtDNA codes for 24 RNAs (12S and 16S ribosomal RNA, 22 tRNAs), and 13 protein subunits of the respiratory chain complexes. Therefore, the subunits of the respiratory complexes are encoded both by nuclear and mitochondrial DNA with the exception of complex II encoded only by the nuclear genome. Other mitochondrial proteins (about 1500) are coded by nuclear DNA and delivered to mitochondria by a localization signal in the amino-terminus of the polypeptides. The most variable part of the DNA molecule is the noncoding region (D-loop region), which is 1.1 kbp long and comprises regulatory regions implicated in mtDNA replication and transcription [7]. MtDNA is located near the mitochondrial respiratory chain—the major source of ROS in the cell—therefore it is more affected by mutations (point mutations and deletions) induced by ROS than nuclear DNA [8]. Some mutations are deleterious to cells because they result in mitochondrial dysfunction, others have no important functional consequences and are considered neutral polymorphisms. However, it is important to know the percentage of deleterious mtDNA mutations (threshold) that can lead to a dysfunction of the mitochondrial respiratory apparatus because it is reported that a very high mutation load may have phenotypic effect [9,10,11].

In reference to neutral polymorphisms, their de novo sequential accumulation in a single mtDNA molecule generates a mtDNA haplotype. A group of related haplotypes gives rise to haplogroups, which can be specific for ethnic groups or geographic areas [12]. Haplogroups may be related to the individual predisposition to diseases [13].

To ensure maximal mitochondrial function, the mitochondrial quality control systems protect mitochondria from ROS damage at the protein, DNA, and organelle level. At the protein level, mitochondria are protected by antioxidant systems, DNA repair, protein folding and degradation. At organelle level, damage activates mitochondrial biogenesis, mitochondrial dynamics (fusion and fission) and mitochondrial autophagy, also known as mitophagy [14].

The best-characterized metabolic phenotype of tumor cells is aerobic glycolysis (the so-called Warburg effect) where cancer cells, even in presence of oxygen, metabolize glucose and produce an excess of lactate. Warburg explains this phenomenon by hypothesizing the presence of defects in the mitochondrial respiratory chain compensated by the increase in glycolytic rate [15]. Aerobic glycolysis may have a key role in supporting the biosynthetic programs of the fast-growing tumor cells. However, the Warburg effect is not a consistent feature in all cancer types and in different cell populations, in fact, cancer cells may be glycolytic, partially mitochondrial OXPHOS-dependent or completely OXPHOS-dependent. In recent years, reprogramming of metabolism has emerged as a new hallmark of tumor development [16].

In cancer, several alterations of mtDNA (deletions, point mutations and copy number variation) cause mitochondrial dysfunction [17]. In addition to mutations that directly affect mtDNA, mutations in nuclear genes coding for mitochondrial proteins, such as tricarboxylic acid cycle genes (succinate dehydrogenase, fumarate hydratase, and isocitrate dehydrogenase1 and 2), have been described in cancer [18,19,20,21]. The mutated proteins contribute to tumorigenesis via stabilization of hypoxia inducible factor 1alpha (HIF1α) [22], thereby probably driving the glycolytic shift that depends strictly on this transcription factor.

Endometrial carcinoma (EC) is one of the most frequently occurring gynecological malignancies in the Western world whose incidence has increased significantly during the last few decades. Endometrioid carcinoma (type I, estrogen-dependent) is the most common form; it displays a less aggressive behavior than type II. The development of type I EC is correlated with unopposed endogenous estrogen exposure; risk factors are chronic anovulation, tamoxifen treatment, nulliparity, early age of menarche, and late age of menopause, age, high body mass index, hypertension, type II diabetes [23]. The unopposed estrogenic stimulation is considered at the basis of a slow progression from normal endometrium to hyperplasia and endometrial cancer [24,25,26]. It is known that estrogens exert direct and indirect effects on mitochondrial function by differential expression and localization of estrogen receptors [27].

In this review, we will provide an overview and update from our previous review [26] on the mitochondrial alterations in type I EC and in endometrial hyperplasia. Moreover, we will highlight the involvement of mitochondrial DNA mutations and respiratory complex I deficiency in activating mitochondrial quality control systems and the role of these mitochondrial alterations in oncogenesis and tumor progression.

## 2. MtDNA Mutations in EC Development and Progression

MtDNA mutations have been described in almost all types of cancer and could play different roles in tumor development and progression.

MtDNA mutation could arise either in the female germ line (germline mutations), and may predispose to cancer, or in the affected tissues, representing tumor-specific somatic mutations. Tumor-specific somatic mtDNA mutations may contribute to tumor development and progression as drivers or as complementary gene mutations according to the multiple-hit model [28]. In particular, they can be tumorigenic or adaptive mutations. Pathogenic mtDNA mutations in genes coding subunits of the mitochondrial respiratory complexes may be tumorigenic, since they may lead to dysfunction in the mitochondrial respiratory chain and may stimulate ROS production. ROS in turn may induce mutations in genes that regulate cell replication in proto-oncogenes and in tumor-suppressor genes, leading to cancer development. Adaptive mutations may be mild mtDNA mutations that may participate in metabolic remodeling and may influence tumor progression conferring to cancer the ability to metastasize [29]. However, some mtDNA mutations may be casually present in cancer, thus having no role in cancer development and progression.

Initial studies describing mtDNA mutations in EC did not distinguish between type I and type II. However, most of the analyzed EC samples were of type I. These mutations included deletions, insertions and point mutations and were located in the D-loop region, or in regions coding for rRNAs, tRNAs, or subunits of mitochondrial respiratory complexes.

Germline mtDNA mutations were investigated in EC to evaluate whether they have the potential to represent markers for predicting the risk of developing EC. MtDNA mutations that predispose or protect from EC are reported in Table 1. Base change (m.16189T>C) located in the D-loop region was associated with susceptibility to EC [30]. A mitochondrial polymorphism associated to haplogroup D (m.5178A>C) in the *ND1* gene was reported to predispose to EC in a southwest Chinese population [31]. Three polymorphisms (m.16223C>A, m.207G>A and m.16126T>C) located in the D-loop region of mtDNA, were associated with an increased risk of EC in the Polish population, whereas the polymorphism m.7028C>T located in the *COI* gene seemed to be a EC protective factor [32].

However, these studies lack functional proof that these common polymorphisms are really able to predispose to EC. Therefore, to exclude a mere association between the above reported mtDNA variants and EC, it would be very interesting to monitor the daughters of these patients harboring these mtDNA polymorphisms to verify whether this risk is increased in the generations to whom the mutation is passed.

Studies on somatic mtDNA mutations in EC have attempted to demonstrate their role as possible molecular markers for cancer detection. In Table 2, somatic mtDNA mutations found in EC are reported.

Some somatic mtDNA mutations are changes in length of short base-repetitive sequences of mtDNA (mitochondrial microsatellite instability, mtMSI) located in the D-loop region and in the 12S rRNA gene [33,35,36,37]. Interestingly, the occurrence of these mtMSI was significantly higher in EC (48.4%) than in breast (29.4%), ovarian (21.9%), and cervical (25.4%) cancer [35]. However, the sequencing of mtDNA of cells isolated from different areas of EC and from adjacent normal tissue by laser-capture microdissection, demonstrated that mtDNA mutations occurred randomly and independently in single cells [34]. Therefore, the authors suggested that it is very unlikely that mtDNA mutations may be involved in EC development, but they may be a secondary event during tumor progression.

Moreover, in endometrial hyperplastic and cancer tissues part of the D-loop region, 16S rRNA, tRNAs and the *ND4L* gene were analyzed by single-strand conformation polymorphism (SSCP) technique to study the incidence of mtDNA mutations [40]. Somatic mtDNA mutations were found in 10% of analyzed patients, however, they were not detected in hyperplastic endometrial tissues. When the relationship between somatic mtDNA mutations and clinical and pathological variables (age, clinical stage, histological grade and type or depth of myometrial invasion) of women affected by EC were studied, no correlation was found.

A more informative picture of the role of mtDNA mutations in cancer development and progression was achieved through the sequencing of the entire mtDNA molecule in type I EC samples and in matched typical hyperplastic samples as control [39]. Tumor-specific mtDNA mutations, identified only in endometrial cancer tissue and not in matched endometrial control tissue, were found in 69% of the analyzed EC patients. Many of these mutations were located in complex I genes, predicted to be pathogenic by *in silico* analysis and had not been previously reported in the literature. Interestingly, pathogenic mutations were absent in hyperplastic tissues and all mtDNA variants detected in hyperplasia were haplogroup determinants. No correlation between the occurrence of tumor-specific mtDNA mutations and clinical data was found, even if low-grade (G1–G2) tumors harbor more pathogenic mtDNA mutations than high-grade (G3) tumors [39].

We have suggested that estrogen may favor the appearance of mtDNA mutations in EC by two mechanisms [26]: (1) estrogen increases mitochondrial ROS [41] that may directly damage mtDNA; (2) estrogen stimulates mitochondrial biogenesis [27] that may cause excessive mtDNA replication and consequently mutations, since mitochondrial DNA polymerase is prone to insert incorrect bases during replication. Accordingly, the mtDNA mutational pattern seems to be related more to mtDNA replication errors than to mutagenic agents in human tumors [42].

The mechanism through which mtDNA mutations are selected and accumulated in cancer cells is still debated; it is likely they expand under the selective pressure of the tumor microenvironment, suggesting they may confer a selective advantage to cancer cells, or they may be subjected to a relaxed selection [43]. In type I EC tissue, but not in hyperplasia, mtDNA mutations may reach detectable values, probably due to these mechanisms, and therefore have the potential to become useful biomarkers for the distinction of tumor versus hyperplastic tissues.

Tumor-specific mtDNA mutation could be an additional diagnostic tool to reveal synchronous nature of simultaneously detected endometrial and ovarian cancer [38,44]. A comparison of tumor-specific mtDNA mutations present in endometrial and ovarian cancer tissues of the same patient would allow us to understand the origin of the two cancers. Since it is improbable the same somatic mutation may occur synchronously and independently in EC and ovarian cancer, the presence of the same tumor-specific mtDNA mutations in both tissues suggests these mutations have a common clonal origin and that one of these cancers is the metastasis of the other.

Nuclear genes commonly involved in progression from hyperplasia to tumor (*KRAS*, *PTEN*, *TP53* and *CTNNB1*) were screened for point mutations in the hyperplastic and tumor samples of the same patients in order to place mtDNA mutations in the EC tumor progression model [39]. About 39% of tumor samples harbored point mutations in the *PTEN* gene. In two cases, the mutation was also detected in the matching hyperplastic tissue, suggesting an early inactivation. Mutations in *KRAS*, *TP53*, and *CTNNB1* genes were found only in tumor samples and not in hyperplastic tissues. Since mtDNA mutations were identified in 69% of cases, while mutational events in nuclear analyzed genes occurred in 56% of the cases, the authors suggested that mtDNA mutations may precede the genetic instability of these genes. The ROS increase, due to mtDNA mutations, may be responsible for nuclear DNA damage and may induce genetic instability and tumor development. However, the authors pointed out that even if a high percentage of EC patients harbor tumor-specific mtDNA mutations, several tumor-specific mtDNA mutations were not potentially pathogenic and finally that not all mutations were homoplasmic or had a high mutation load that imply a mitochondrial dysfunction [39].

Therefore, the role of tumor-specific mtDNA mutation in EC is still a matter of controversy: although it seems likely that they contribute to cancer, inducing nuclear DNA damage, they may be merely a side effect of tumorigenesis. To address this topic more research is needed.

## 3. Deficit of Respiratory Complex I in Type I EC

The main site of energy production in cells, the mitochondrial oxidative phosphorylation system (OXPHOS), is localized in the inner membrane of mitochondria. OXPHOS machinery is composed of four complexes (complex I, II, III, and IV) responsible for electron transport and proton translocation and for the adenosine triphosphate (ATP) synthase complex (complex V). From Warburg’s observations at the beginning of the last century to the most recent research, the role of the OXPHOS system and, in particular, of respiratory complex I (CI) emerges as central in cancer development and progression [22,43]. CI is the largest complex, being composed of 44 subunits, seven of which (ND1-6 and ND4L) are encoded by mtDNA.

A disassembly of CI has been demonstrated in oncocytomas, tumors characterized by mitochondria hyperproliferation (oncocytic-like foci) and by high load of pathogenic tumor-specific mtDNA mutations (nonsense and frameshift) in CI [45,46,47]. These results suggested the altered mitochondrial function due to mtDNA mutations can be compensated by mitochondrial hyperproliferation. Also in type I EC, most EC samples, characterized by the presence of pathogenic tumor-specific mtDNA mutations, showed oncocytic-like foci and a partial or total loss of immunohistochemical staining for the ND6 subunit of complex I in some of them, suggesting a deficit of CI [39]. Recently, CI has been investigated in two type I EC patients [48] by nondenaturing Blue Native Polyacrylamide Gel Electrophoresis (BN-PAGE) and enzymatic colorimetric reactions, confirming a deficit of CI activity in cancer samples compared to matched controls. Western blotting analysis on respiratory complexes separated by BN-PAGE with antibodies against subunits of respiratory complexes I, IV and II showed a decrease in CI amount. These results confirmed an association in type I EC between pathogenic mtDNA mutation, loss of CI, and oncocytic-like transformation as already reported in oncocytomas. The mtDNA mutations in complex I genes associated to deficit of CI are reported in Table 3.

It has been suggested CI can be considered an “*oncojanus*” [43]. Mild CI dysfunction may contribute to tumor metabolism and to tumorigenic properties of cancer cells enhancing oxidative stress and activating the oncogenic Akt/mTORC1 pathway. Conversely, since cancer cells are characterized by a high-energy demand for proliferation, the severe CI defects in oncocytomas may induce a metabolic short-circuit preventing tumor progression, thus leading to an almost benign phenotype.

Therefore, it can be envisioned that also in EC a combined action of estrogens and complex I dysfunction may contribute to maintain the tumor in a less aggressive state and can explain how type I EC prognosis is generally more favorable. However, the functional role of CI dysfunction in EC deserves in-depth investigation.

## 4. Mitochondrial Biogenesis Increase in Hyperplasia and Type I EC

Changes in mitochondrial number, mtDNA content and mRNA expression for OXPHOS genes have been reported in solid tumors [49].

The master regulator of mitochondrial biogenesis is the nuclear transcriptional coactivator belonging to PPARγ coactivators (PGC) family, namely PPARγ-coactivator-1 alpha (*PGC-1α*) [50]. PCG1-α is a coactivator of nuclear respiratory factors 1 and 2 (*NRF-1* and *NRF-2*) and by means of these factors enhances the expression of many nuclear genes, in particular, that of the mitochondrial transcription factor A (*TFAM*), which is a key factor in regulating mtDNA transcription and replication. [50].

An increase in TFAM, NRF-1 and PGC-1α protein content was found in a pooled group of type I EC endometrial tissues compared with a pooled group of endometrial proliferative control tissue suggesting, in type I EC tissue, an upregulation of the PGC-1α signaling pathway and an increase in mitochondrial biogenesis [51]. The increase in mitochondrial biogenesis is generally measured by an increase in the mtDNA/nuclear DNA ratio (mtDNA cellular content) and in citrate synthase (CS) activity (marker of mitochondrial mass). In fact, the mtDNA cellular content was measured in EC cells collected by laser-capture microdissection revealing a twofold increase in EC compared with normal endometrial cells [52]. Moreover, a twofold increase in mtDNA content and in CS activity was found in a pooled group of type I EC endometrial tissues compared to a pooled group of endometrial proliferative control tissue [51]. An increase in mtDNA content in structural mitochondrial proteins TFAM and voltage-dependent anion channel 1 (VDAC1). In some nuclear DNA-encoded respiratory subunits NADH:ubiquinone oxidoreductase subunit A9 (NDFUA9), succinate dehydrogenase complex flavoprotein subunit A and B (SDHA, SDHB), Core II was also found in EC samples compared to matched control tissues, especially, in the EC samples harboring pathogenic tumor-specific mtDNA mutations. In 72% of these analyzed patients, oncocytic-like foci were also found confirming the association between mtDNA mutations and the increase in mitochondrial biogenesis in type I EC [39]. Different results were found by Reznick et al. [49] reporting a decrease of OXPHOS mitochondrial genes expression and no increase of mtDNA in EC compared to adjacent-normal tissue.

The mtDNA content and CS activity were also measured in control, hyperplastic (with or without atypia) and cancer endometrial tissues to verify if they could be considered possible markers for progression from benign to premalignant lesions [53]. This analysis revealed an increase in mtDNA content in hyperplasia and, in particular, in ECs compared with controls. The same trend was found for CS activity. These data also revealed that an mtDNA content increase preceded the increase in CS activity, since a statistically significant increase was observed for mtDNA content already in typical hyperplasia, while an analogous increase for CS activity was found only in atypical hyperplasia. No statistically significant correlation was found between the mtDNA content or CS activity and prognostic factors (grade, depth of myometrial invasion, stage). However, in high-grade tumors, mtDNA content was slightly decreased, probably due to the high rate of cell division and the consequent lower number of mitochondria per cell and to the lower estrogen exposure.

Estrogen has an important role in mitochondrial homeostasis. The genomic activity of estrogen is mediated by estrogen receptors (ERα and ERβ). They have been identified in different cell compartments and also in cell-type-dependent manner colocalize within mitochondria. They stimulate mitochondrial biogenesis by activating *NRF-1* transcription and, by directly interacting with D-loop, increasing mtDNA transcription [27,54,55,56]. In particular, in breast and lung adenocarcinoma estradiol, by stimulating directly *NRF-1* that increase *TFAM* genes expression, enhanced mitochondrial biogenesis and oxygen consumption [57]. Moreover, in breast cancer cells, estradiol increased mitochondrial ROS, which stimulated NRF-1 activity [58].

Estradiol regulates also ion homeostasis increasing intracellular Calcium uptake, expression of the antiapoptotic factor *Bcl-2*, which augments the maximal mitochondrial calcium uptake capacity [59]. Increased intracellular calcium, through activation of Calcium dependent protein kinases and phosphatase, may regulate corepressors and coactivators as PGC-1α modulating gene transcription [27]. Calcium can cause also changes in cellular function and may contribute to cancer progression and metastasis [60].

Therefore, it can be envisioned that the moderate increase in mitochondrial biogenesis reported in endometrial hyperplasia could be a direct result of estrogen stimulation. In type I EC cancer tissues, the effect of estrogen stimulation on the increase in mitochondrial biogenesis may be reinforced by the occurrence of pathogenic tumor-specific mtDNA mutations. These mutations, in fact, may lead to respiratory dysfunction and ROS increase, thus triggering a retrograde signaling to the nucleus, through the upregulation of the PGC-1α signaling pathway [26].

Since mtDNA content and CS activity increased in cancer and also in atypical hyperplasia it could be envisioned that they represent possible molecular markers to establish the risk of malignant transformation in endometrial hyperplasia and may have a clinical value in patient management. However, due to the high interindividual variability of these markers, further analysis in a wider panel of patients and prospective longitudinal studies is necessary to address this topic.

## 5. Activation of Antioxidant Response in Type I EC

In cancer, oxidative stress condition induces an increase in antioxidant enzymes as a compensatory defensive mechanism to counteract ROS increase and to maintain mitochondrial function [61].

It is plausible to hypothesize that oxidative stress conditions may occur in EC, since estrogens and mitochondrial respiratory dysfunction may increase ROS production in mitochondria, and may activate the mitochondrial redox defense system. This system consists of antioxidant proteins namely peroxiredoxin 3 (Prx3), peroxiredoxin 5 (Prx5), manganese superoxide dismutase, and thioredoxin 2. These proteins eliminate ROS that have been generated in the oxidative phosphorylation system. Indeed, an increase in Prx3, manganese superoxide dismutase and catalase was reported in type I EC, especially in patients harboring pathogenic tumor-specific mtDNA mutations [39]. Moreover, an increase in Prx3 and Prx5 was also reported in endometrial cancer [62] and Prx3 was found upregulated in EC cells and in endometrial cancer stem cells (CSCs) [63]. The knockdown of the Prx3 gene in these endometrial CSCs resulted in the death of cells by causing mitochondrial dysfunction. This result indicated that Prx3 eliminated ROS and it was required for the maintenance of mitochondrial function and the survival of CSCs.

Furthermore, an increase in the expression level of augmenter of liver regeneration (ALR) protein was recently reported in type I EC [48]. It was reported that, in other tissues, ALR has an antioxidant activity, stimulates mitochondrial biogenesis [64] and, by inducing the antiapoptotic protein Bcl-2, acts as antiapoptotic factor [65,66]. Moreover, in glioma cells, it was demonstrated that ALR had antioxidative activity by reducing ROS and protecting cells from ROS-induced oxidative damage, since it stimulated the expression of clusterin, a reducing agent [67]. Concordantly with these findings, the increase in clusterin mRNA [68] and in Bcl-2 protein [47] was also reported in endometrial cancer suggesting that the increase in ALR might be a protective response to the ROS increase.

Therefore, it can be envisioned that Prx3 and ALR might represent valuable therapeutic targets and could provide new insights into the development of new therapeutic strategies for patients with endometrial cancer.

## 6. Activation of The Mitochondrial Quality Control Systems in Type I EC

It has been suggested that mitochondrial dysfunction in cancer cells may activate mitochondrial quality control systems, such as mitochondrial biogenesis, mitochondrial dynamics (fusion and fission), mitophagy and protein turnover, as a compensatory response [14,69,70].

A marked increase in mitochondrial fission protein Dynamin related protein 1 (Drp1) and a decrease in fusion protein mitofusin protein 2 (Mfn2) were found in type I EC patients, characterized by the deficit of respiratory complex I and oncocytic-like foci, compared with matched controls, suggesting an increase in mitochondrial fission [48]. This analysis was extended to a pooled group of type I EC, of endometrial hyperplasia and of nonmalignant tissues revealing an increase in the mitochondrial fission proteins Drp1 and Fission protein 1 (Fis1) in cancer compared with control and hyperplastic tissues. Mfn2 was also found to be significantly decreased in cancer compared to control and hyperplastic tissues. Moreover, an increase in the expression level of Bcl-2 and adenovirus E1B 19 kDa-interacting protein (BNIP3), the molecular mediator implicated in promoting mitophagy, and in the caseinolytic mitochondrial matrix peptidase proteolytic subunit (CLPP) was also observed. These results suggested that, not only the increase in mitochondrial biogenesis, but also fission, mitophagy and proteolysis may be activated in type I EC to ensure a sufficient number of functional mitochondria to survive mitochondrial dysfunction better [48].

A key question is how mitochondrial dysfunction might regulate mitochondrial dynamics to facilitate a fragmented mitochondrial network. It has been suggested that oncogenic K-Ras, via Extracellular signal-regulated kinase 1 and2 (ERK1/2)-mediated phosphorylation of Drp1, promotes mitochondrial fragmentation and forces cellular metabolism towards glycolysis [71]. Supporting this hypothesis, an increase in phosphorylated Drp1 on serine 616 [48] and heterozygous mutations in the critical amino acids of K-Ras [39] were detected in type I EC, suggesting an increase in mitochondrial fragmentation via the K-Ras pathway.

The clinical utility of mitochondrial dynamics, biogenesis and mitophagy as biomarkers for cancer progression is only at the beginning and requires substantial future efforts.

## 7. Conclusions

Estrogens may have a role in type I EC development through direct and indirect effects on mitochondrial function.

**Figure 1:**
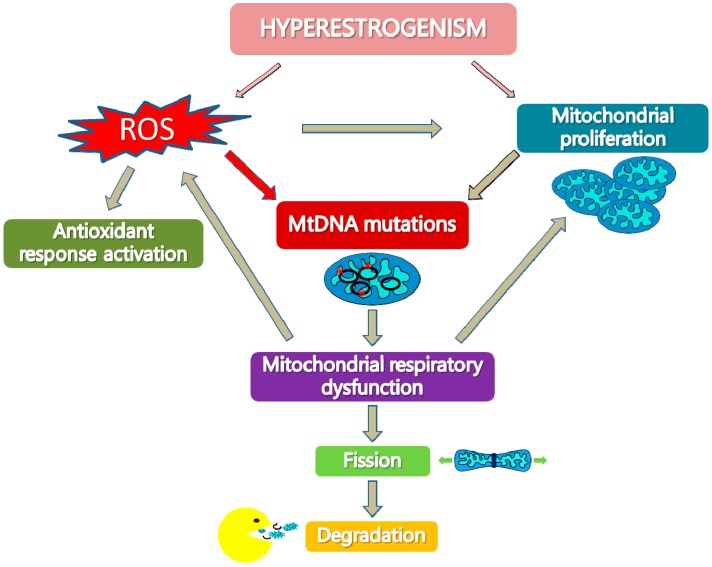
Hyperestrogenism and mitochondrial dysfunction in type I endometrial cancer. Hyperestrogenism may stimulate reactive oxygen species (ROS) production and increase mitochondrial biogenesis. ROS increase and excessive mitochondrial DNA (mtDNA) replication due to increased mitochondrial biogenesis may lead to mtDNA mutations. These mutations may affect respiratory complexes, in particular complex I, and may induce mitochondrial dysfunction that reinforces ROS production and stimulates mitochondrial proliferation in a vicious cycle. ROS increase activates the antioxidant response. Mitochondrial dysfunction may also increase mitochondrial fission in order to segregate damaged mitochondria components, which can then be degraded by proteolysis and mitophagy.

We propose (Figure 1) that hyperestrogenism may stimulate ROS production and may increase mitochondrial biogenesis because of a direct interaction of estrogens with NRF-1 and of ROS activation of NRF-1. Estrogen-related ROS increase and excessive mtDNA replication, due to increased mitochondrial biogenesis, may lead to an increase in tumor-specific mtDNA mutations. These mutations may reach a threshold value and affect respiratory complexes, in particular complex I, and may lead to respiratory dysfunction and ROS increase. Mitochondrial dysfunction and ROS increase, in a vicious cycle, may in turn reinforce the occurrence of mtDNA mutations and trigger a retrograde signaling to the nucleus that stimulates further mitochondrial proliferation and activates antioxidant response as a compensatory mechanism. Moreover, as an adaptation process to mitochondrial dysfunction, mitochondrial fission may be stimulated in order to segregate damaged mitochondria components that can be discharged by proteolysis and mitophagy.

We have also highlighted the fact that pathogenic mtDNA mutations are hallmarks of EC and are potentially useful tools for tumor diagnosis and prognosis. They could be useful biomarkers for the distinction of tumor versus hyperplastic tissues, since they are present in high percentages only in type I EC, they can be markers of low-grade tumors. Moreover, mtDNA mutations may provide an additional diagnostic tool to reveal synchronous cancers.

A key unresolved question is whether tumor-specific mtDNA mutations may play a role in oncogenesis and tumor progression processes, through ROS increase and genetic instability, or whether they are merely a side effect of tumorigenesis. Certainly, they could provide an explanation for altered mitochondrial phenotype and for the activation of mitochondrial quality control systems in EC.

There are grounds to state that within the field of studies on EC, future studies on mtDNA mutations and on the expression level of proteins involved in altered mitochondrial pathways should be implemented, since they could open new horizons in the diagnosis and in the prognosis of EC and, most importantly, could represent potential therapeutic targets.

## Figures and Tables

**Table 1:** Germline mitochondrial DNA mutations that may predispose or protect from endometrial cancer.

Mutation	Gene	Mutation type	Population	Effect	References
m.16189T>C	*D-loop*	Point mutation	Chinese	Predispose	[30]
m.16223C>A	*D-loop*	Point mutation	Polish	Predispose	[32]
m.207G>A	*D-loop*	Point mutation	Polish	Predispose	[32]
m.16126T>C	*D-loop*	Point mutation	Polish	Predispose	[32]
m.5178A>C	*ND2*	Point mutation	Chinese	Predispose	[31]
m.7028C>T	*COI*	Point mutation	Polish	Protect	[32]

Abbreviations: m., mitochondrial.

**Table 2:** Somatic mitochondrial DNA mutations in endometrial cancer.

Mutation	Gene	Mutation type	AA	References
m.152T>C	*D-loop*	Point mutation	-	[33]
m.251G>A	*D-loop*	Point mutation	-	[33]
m.294T>C	*D-loop*	Point mutation	-	[33]
m.289-346del	*D-loop*	50bp deletion	-	[33]
m.305C>A	*D-loop*	Point mutation	-	[34]
m.306C>G	*D-loop*	Point mutation	-	[34]
m.303-309	*D-loop*	mtMSI	-	[33,34,35,36,37]
m.309C>A	*D-loop*	Point mutation	-	[34]
m.514-523	*D-loop*	mtMSI	-	[33,34,35,37]
m.16153G>A	*D-loop*	Point mutation	-	[32]
m.16182A>C	*D-loop*	Point mutation	-	[34]
m.16183A>C	*D-loop*	Point mutation	-	[34]
m.16184-16193	*D-loop*	mtMSI	-	[33,34,35,37]
m.16188A>C	*D-loop*	Point mutation	-	[32]
m.16189T>C	*D-loop*	Point mutation	-	[34]
m.650T>C	*12S rRNA*	Point mutation	-	[33]
m.817G>A	*12S rRNA*	Point mutation	-	[33]
m.879T>C	*12S rRNA*	Point mutation	-	[33]
m.956-965	*12S rRNA*	mtMSI	-	[33,34,35,37]
m.961T>C	*12S rRNA*	Point mutation	-	[34]
m.1474G>A	*12S rRNA*	Point mutation	-	[38]
m.3163G>A	*16S rRNA*	Point mutation	-	[33]
m.3470T>Y	*ND1*	Point mutation	L55P	[39]
m.3730T>Y	*ND1*	Point mutation	Y142H	[39]
m.3670G>A	*ND1*	Point mutation	A122T	[39]
m.3425T>Y	*ND1*	Point mutation	V40A	[39]
m.4722A>G	*ND2*	Point mutation	Y85A	[38]
m.5212T>C	*ND2*	Point mutation	L248P	[39]
m.5567T>C	*TW*	Point mutation	-	[38]
m.6129G>R	*COI*	Point mutation	G76stop codon	[39]
m.6562T>C	*COI*	Point mutation	F220S	[39]
m.6822T>A	*COI*	Point mutation	S307T	[39]
m.6991T>Y	*COI*	Point mutation	L363P	[39]
m.7962T>Y	*COII*	Point mutation	L126S	[39]
m.8545G>A	*ATP6*	Point mutation	A7T	[39]
m.10290G>A	*ND3*	Point mutation	A78T	[39]
m.11863insC	*ND4*	Point mutation	-	[38]
m.11873insC	*ND4*	Point mutation	-	[38]
m.12425insA	*ND5*	Point mutation	-	[38]
m.12439T>C	*ND5*	Point mutation	Y35H	[39]
m.13718G>A	*ND5*	Point mutation	S461N	[39]
m.13994T>C	*ND5*	Point mutation	L553P	[38]
m.14279G>A	*ND6*	Point mutation	S132L	[39]
m.14510delA	*ND6*	Point mutation	-	[38]
m.15172G>A	*CYB*	Point mutation	S	[38]
m.15247C>T	*CYB*	Point mutation	S	[38]
m.15573T>C	*CYB*	Point mutation	F276S	[38]
m.15831T>C	*CYB*	Point mutation	I362T	[39]

Abbreviations: mtMSI, mitochondrial microsatellite instability; AA, aminoacidic change; -, no change; S, synonymous mutation.

**Table 3:** Mitochondrial DNA mutations in complex I genes associated to deficit of complex I.

Mutation	Gene	Mutation type	AA	References
m.3730T>Y	*ND1*	Point mutation	Y142H	[39,48]
m.3425T>Y	*ND1*	Point mutation	V40A	[39,48]
m.5212T>C	*ND2*	Point mutation	L248P	[39]
m.10844A>C	*ND4*	Point mutation	T29P	[39]
m.14510delA	*ND6*	Point mutation	-	[39]

Abbreviations: AA, aminoacidic change; -, no change.
